# Independent effects of sham laparotomy and anesthesia on hepatic microRNA expression in rats

**DOI:** 10.1186/1756-0500-7-702

**Published:** 2014-10-08

**Authors:** Wiebke Werner, Hannes Sallmon, Annekatrin Leder, Steffen Lippert, Anja Reutzel-Selke, Mehmet Haluk Morgül, Sven Jonas, Christof Dame, Peter Neuhaus, John Iacomini, Stefan G Tullius, Igor M Sauer, Nathanael Raschzok

**Affiliations:** General, Visceral, and Transplantation Surgery, Experimental Surgery and Regenerative Medicine, Charité - Universitätsmedizin Berlin, Campus Virchow Klinikum, Berlin, Germany; Department of Neonatology, Campus Virchow Klinikum, Charité - Universitätsmedizin Berlin, Berlin, Germany; Department of Visceral, Transplantation, Thoracic, and Vascular Surgery, Universitätsklinikum Leipzig, Leipzig, Germany; Molecular Cardiology Research Institute, Tufts University School of Medicine and Tufts Medical Center, Sackler School of Biomedical Sciences, Boston, USA; Division of Transplant Surgery and Transplant Surgery Research Laboratory, Brigham and Women’s Hospital and Harvard Medical School, Boston, USA

**Keywords:** Hepatectomy, Liver regeneration, microRNA, Sham laparotomy, Surgical stress

## Abstract

**Background:**

Studies on liver regeneration following partial hepatectomy (PH) have identified several microRNAs (miRNAs) that show a regulated expression pattern. These studies involve major surgery to access the liver, which is known to have intrinsic effects on hepatic gene expression and may also affect miRNA screening results. We performed two-third PH or sham laparotomy (SL) in Wistar rats to investigate the effect of both procedures on miRNA expression in liver tissue and corresponding plasma samples by microarray and qRT-PCR analyses. As control groups, non-treated rats and rats undergoing anesthesia only were used.

**Results:**

We found that 49 out of 323 miRNAs (15%) were significantly deregulated after PH in liver tissue 12 to 48 hours postoperatively (>20% change), while 45 miRNAs (14%) were deregulated following SL. Out of these miRNAs, 10 miRNAs were similarly deregulated after PH and SL, while one miRNA showed opposite regulation. In plasma, miRNA upregulation was observed for miR-133a and miR-133b following PH and SL, whereas miR-100 and miR-466c were similarly downregulated following anesthesia and surgery.

**Conclusions:**

We show that miRNAs are indeed regulated by sham laparotomy and anesthesia in rats. These findings illustrate the critical need for finding appropriate control groups in experimental surgery.

## Background

The liver is a remarkable organ, not only with respect to its diverse functions, but also its unique regenerative capacity. After surgical resection, the liver is capable of fully restoring its lost mass by compensatory growth of the remaining tissue [1, 2]. Partial hepatectomy (PH) is a common treatment for primary and secondary malignancies of the liver [3, 4]; therefore, it is important to fully understand the process of liver regeneration. It is already well known that cytokines, growth factors, and the metabolic network initiate and modulate liver regeneration, but the underlying mechanisms that govern this regulation are not completely understood [5]. As part of the posttranscriptional regulatory network, the role of small, non-coding microRNAs (miRNAs) during liver regeneration after PH has recently been investigated. These studies have shown that miRNAs are essential key players in the initiation, modulation, and termination of liver regeneration [6–14].

PH is associated with major surgery to access the liver, triggering intrinsic side effects, such as local tissue damage, inflammation, and wound healing. Thus, sham surgery control groups are essential to control for effects caused by the surgical procedure, especially considering that these procedures also modulate the global gene expression of non-treated healthy livers [15–17]. For example, Juskeviciute et al. performed cDNA microarrays on the liver tissue of partially hepatectomized and sham-operated rats and found 16 genes (e.g., Lzts2 and Serpina10) to be significantly regulated by sham surgery compared to non-treated liver tissue [15]. Dransfeld et al. found that the expression of 16 out of 242 investigated genes was affected during the period of 48 hours after a sham operation, e.g. downregulation of the organic anion transporting polypeptide (Oatp)2 and upregulation of Oatp5 [16]. Interestingly, four genes (growth responsive protein, Oatp5, p47phox, and system A (ATA2)) were even more affected by sham surgery than by PH. Thus, when interpreting these studies, the effects of surgical procedures on hepatic and global gene expression should be carefully taken into account. Similar effects may lead to false-positive or confounding results of miRNA screenings after PH.

Based on these considerations, we investigated the effect of sham surgery and anesthesia on microRNA expression profiles in the liver. We analyzed miRNA expression patterns in the liver of sham-operated rats and compared our findings with miRNA analyses performed on liver tissue of rats after 70% PH and after anesthesia only. Additionally, we measured expression levels of specific miRNAs in the plasma of these animals.

## Methods

### Animal studies

#### Sham laparotomy versus partial hepatectomy in Wistar rats

All experiments were performed after approval of the Berlin institutional review board (license no. G 0154/09). Male Wistar rats with an average weight of 320 g were randomly divided into three groups (n = 3 per group and time point). The first group (PH-group) underwent 70% partial hepatectomy according to Higgins and Anderson [18] as previously described [6]. The animals in the second group underwent sham surgery consisting of anesthesia, medial ventral laparotomy, manual palpation of the liver, and analgesic medication (SL-group). The total operation time for both groups was approximately 45 minutes. As controls, animals treated only with anesthesia and analgesic medication (AN-group) and non-treated animals were used.

The animals in the AN-group were treated with the same anesthesia protocol used during PH and sham operations. Briefly, animals were anesthetized for five minutes using 4.0 vol.% of isoflurane, and settled into an anesthesia mask and treated with 2.5 vol.% for 30 minutes and 1.5 vol.% for another ten minutes. For perioperative pain treatment, the animals of the PH- and SL-group received a single subcutaneous injection of buprenorphine (Temgesic® 0.01 mg/kg body weight) thirty minutes before the end of surgery, while the animals of the AN-group received an injection of metamizole (Novaminsulfon® 50 mg/kg body weight). Additionally, all animals had free access to metamizole (50 mg/kg body weight) via the drinking water.

Animals in the PH-group and SL-group were sacrificed 12, 24, and 48 hours after the procedure, animals of the AN-group were sacrificed after 24 hours, and non-treated animals (used for normalization) were sacrificed immediately. Briefly, the aorta was punctured following 4.0 vol.% isoflurane narcotization and blood samples were collected in EDTA tubes. Subsequently, the portal vein was cannulized, and the liver was flushed with ice-cold saline (total anesthesia time: 7 minutes). Following liver explantation, tissue samples were immediately snap frozen in liquid nitrogen. EDTA plasma was centrifuged at 1,500 × g for 15 min within 30 minutes after blood collection, and the supernatant plasma was carefully removed and stored in 200 μl aliquots at −80°C until further analysis.

### miRNA screening and validation

#### RNA purification from tissue and plasma samples

For RNA isolation, tissue samples with an average weight of 20 mg were homogenized in 500 μl TRIzol RNA Isolation Reagent (Invitrogen, Carlsbad, CA, USA). Plasma samples (200 μl) were thawed on ice and centrifuged at 12,000 × g for 30 min at 4°C to concentrate cell-free elements such as nucleotides in the lower part of the tube. Next, the upper 150 μl of plasma were removed from the sample and discarded. The remaining 50 μl of plasma were mixed with 500 μl TRIzol RNA Isolation Reagent (1:10 volumes). RNA was isolated from tissue specimens and plasma samples using the same protocol. Briefly, samples were mixed with 100 μl chloroform and centrifuged at 12,000 × g for 15 min at 4°C. The supernatant was collected and mixed with 500 μl isopropanol. After one hour of incubation, the samples were centrifuged, and the isopropanol was carefully removed. After rinsing with 1 ml of 75% ethanol, pellets were left to dry at 56°C and then dissolved in 20 μl DEPC-treated water. The RNA content and 260/280 ratio were assessed using an Agilent 2100 Bioanalyzer (Agilent Technologies, Santa Clara, CA, USA).

#### miRNA microarray screening

The hybridization of individual liver samples (PH-group, SL-group, and non-treated animals) was performed for 16 h at 42°C using the Geniom Biochip MPEA *Rattus norvegicus* (Sanger miRBase version 14.0) from febit Inc. with the Geniom RT®-Analyzer (febit, Heidelberg, Germany) as previously described [6]. The microarray data have been deposited in NCBI’s Gene Expression Omnibus and are accessible through GEO Series accession number GSE23696

#### Quantitative real-time PCR analysis for miRNAs

All products were obtained from Applied Biosystems (Foster City, CA). TaqMan MicroRNA Assays specific for miR-100, miR-105, miR-133a, miR-133b and miR-466c were used. U6 was chosen for normalization of tissue miRNA expression whereas miR-451 was used for normalization of plasma miRNA [19]. miRNA-specific cDNA was synthesized from 100 ng of tissue-derived miRNA or 1 μg of plasma-derived RNA using the TaqMan® MicroRNA Reverse Transcription Kit. PCR amplification was performed using TaqMan® Universal Master Mix II with UNG at 95°C for 10 minutes, followed by 50 cycles of 95°C for 15 seconds and 60°C for 1 minute in a PCR system (Applied Biosystems). Analyses were performed using StepOnePlus™ (Applied Biosystems) in triplicate for tissue miRNAs and in quintuplicate for plasma miRNAs. Relative changes in miRNA expression were determined using the 2^-∆∆Ct^ method.

### Statistical analysis

The miRNA expression in tissue and plasma samples from animals in the PH-, SL-, or AN-groups was normalized to non-treated animals. Statistical analyses (Empirical Bayes Statistics) adjusted for multiple testing, Student’s t-test, and one-way ANOVA with posthoc multiple comparisons were performed using Bioconductor open source software, Microsoft Excel for Mac (V 14.1.4), and GraphPad Prism (Prism 4 for Macintosh Version 4.0c (GraphPad Software, La Jolla, CA). All data are expressed as mean ± standard deviation (SD). A p-value of less than 0.05 was considered statistically significant.

## Results

### MicroRNA expression in the liver changes significantly after partial hepatectomy and sham laparotomy in rats

Global miRNA microarray screening was performed on a total of 323 miRNAs in rat liver samples at 12, 24, and 48 hours after PH or SL using the same tissue samples and microarray data as in our previously published study [6]. In the PH-group, 49 miRNAs were significantly deregulated (e.g., rno-miR-26a/b, rno-miR-125b-5p and various members of the let-7 family), showing an expression change to at least ≤ 0.8 or ≥ 1.2 compared to normal healthy liver [6], while 45 miRNAs showed significant expression changes in liver samples of animals undergoing SL (Table 1). Comparing significantly deregulated miRNAs in the SL- and PH-group, we identified 10 miRNAs (rno-miR-100, rno-miR-105, rno-miR-1224, rno-miR-133a/b, rno-miR-383, rno-miR-466c, rno-miR-483, rno-miR-598-5p, and rno-miR-628) that showed similar expression changes in both groups at the same postoperative time point, while one miRNA (rno-miR-29a) was regulated in the opposite direction at the same time point. Additionally, 34 miRNAs were significantly deregulated due to sham laparotomy but were unaffected following PH (e.g., rno-miR-205, rno-miR-295, rno-miR-337 and rno-miR-708). The five miRNAs that showed the strongest expression changes from each group in Table 1 were included for putative target analysis using the TargetScan rat algorithm (http://www.targetscan.org; Release 6.2, June 2012). Table 2 provides a selection of putative targets.

**Table 1:** **miRNAs differentially expressed in rat liver after PH and comparison with miRNA expression after SL**

miRNA	FC (SL/control)			FC (PH/control)			Accession No.
	12 h	24 h	48 h	12 h	24 h	48 h	
**A miRNAs similarly deregulated after partial hepatectomy and sham laparotomy**							
rno-miR-100	0.72*	0.92	0.86	0.69**	0.70**	0.88	MIMAT0000822
rno-miR-105	1.56*	1.50***	1.49 **	1.49	1.50***	1.61*	MIMAT0012825
rno-miR-133a	0.99	0.76*	0.70*	0.75	0.68**	0.80	MIMAT0000839
rno-miR-133b	0.82	0.68*	0.74	0.88	0.59**	0.84	MIMAT0003126
rno-miR-383	1.16	1.26*	1.25	1.25	1.29*	1.07	MIMAT0003114
rno-miR-466c	1.24	1.33**	1.03	1.36	1.53***	1.28	MIMAT0005279
rno-miR-483	0.78*	0.82***	0.73***	0.60***	0.70***	0.56***	MIMAT0003121
rno-miR-598-5p	1.32	1.41**	1.32	1.46	1.40*	1.24	MIMAT0005324
rno-miR-628	1.29	1.37*	1.22	1.38*	1.31*	1.24	MIMAT0012836
rno-miR-1224	0.72	0.73***	0.79	0.65*	0.69**	0.64*	MIMAT0012827
**B miRNAs with opposite regulation after partial hepatectomy and sham laparotomy**							
rno-miR-29a	1.05	1.24**	1.17	0.91	0.78***	0.85	MIMAT0000802
**C miRNAs with deregulation after sham laparotomy but not following partial hepatectomy**							
rno-miR-23b	0.97	1.21***	1.16*	0.87	0.80*	0.86	MIMAT0000793
rno-miR-30b-5p	1.14	1.22**	1.17	0.88	0.88*	0.90	MIMAT0000806
rno-miR-31	1.16	1.21***	1.24*	0.95	0.92	0.85	MIMAT0000810
rno-miR-92b	0.70*	0.75**	0.66***	0.87	0.92	0.89	MIMAT0005340
rno-miR-129	0.81	0.86	0.77*	1.03	0.86	0.87	MIMAT0000600
rno-miR-143	1.27	1.32***	1.38*	1.06	0.98	1.00	MIMAT0000849
rno-miR-151	1.13	1.21**	1.18*	0.91	0.93	0.91	MIMAT0000614
rno-miR-191	1.12	1.27***	1.22*	0.98	0.96	1.04	MIMAT0000866
rno-miR-192	1.15	1.21**	1.18	0.94	0.87**	0.95	MIMAT0000867
rno-miR-194	1.17	1.22**	1.20	0.93	0.88**	0.96	MIMAT0000869
rno-miR-195	1.18	1.21***	1.17*	0.95	0.87	0.92	MIMAT0000870
rno-miR-201	1.33*	1.30**	1.30*	1.31	1.18	1.11	MIMAT0012846
rno-miR-205	0.75	0.68**	0.76*	0.94	0.97	0.92	MIMAT0000878
rno-miR-224	1.12	1.28	1.46*	1.06	1.14	1.40	MIMAT0003119
rno-miR-295	0.82	0.67**	0.69*	0.99	0.91	0.89	MIMAT0012849
rno-miR-296	0.78	0.77*	0.76	0.84	0.86	0.89	MIMAT0004742
rno-miR-324-5p	0.77	0.76**	0.77*	0.91	0.89	0.92	MIMAT0000553
rno-miR-331	0.76	0.81	0.70*	0.79	0.71*	0.87	MIMAT0000570
rno-miR-337	1.12	1.42**	1.34	1.14	1.23	1.45	MIMAT0000577
rno-miR-343	0.70*	0.73*	0.68*	0.78	0.81	0.85	MIMAT0000591
rno-miR-365	0.66*	0.83*	0.76*	0.85	0.87	0.80	MIMAT0001549
rno-miR-425	1.14	1.21*	1.27*	1.14	1.05	1.12	MIMAT0005314
rno-miR-429	0.91	0.74**	0.81	0.95	0.91	0.81	MIMAT0001538
rno-miR-484	0.75*	0.66***	0.61***	0.89	0.81*	0.71	MIMAT0005319
rno-miR-511	0.94	1.32	1.47*	0.98	0.99	1.12	MIMAT0012829
rno-miR-532-5p	0.99	1.22*	1.22	1.05	0.98	1.05	MIMAT0005322
rno-miR-615	0.78	0.71*	0.87	0.93	0.92	0.96	MIMAT0012835
rno-miR-664	0.88	0.83	0.74*	0.81	0.73*	0.89	MIMAT0003382
rno-miR-667	0.81	0.85**	0.79**	0.84	0.83	0.84	MIMAT0012852
rno-miR-668	0.80	0.73**	0.85	0.94	0.90	0.87	MIMAT0012839
rno-miR-708	1.55*	1.37*	1.47*	1.25	1.21	1.18	MIMAT0005331
rno-miR-760-3p	0.67*	0.81	0.81	1.08	1.05	0.87	MIMAT0005337
rno-miR-760-5p	0.78*	0.73***	0.74**	0.89	0.84	0.88	MIMAT0005336
rno-miR-880	0.91	0.74*	0.94	0.87	0.81	0.78	MIMAT0005288

miRNAs listed in this table were significantly deregulated at the same time point after sham laparotomy or after partial hepatectomy in rat liver tissue (<0.8 fold change or > 1.2 fold change compared to normal rat tissue; Empirical Bayes Statistics adjusted for multiple testing, each time point compared with 0 h; *p < 0.05, **p < 0.01, ***p < 0.001). Boldface indicates miRNA deregulation at the same postoperative time point. *Abbreviations:* *FC* fold change, *SL* sham laparotomy, *PH* partial hepatectomy.

**Table 2:** **TargetScan analysis of the most deregulated miRNAs**

	#	miRNA	Strongest expression change	Putative targets
**A. miRNAs similarly deregulated following PH and SL**	1	rno-miR-105	1,61	N/A
	2	rno-miR-466c	1,53	HLF, NFIB, HHIP
	3	rno-miR-483	0,56	IGF1, SMAD4, DLC1
	4	rno-miR-133b	0,59	SP3, MAP3K3, FOXC1
	5	rno-miR-598-5p	1,4	N/A
**B. miRNAs with opposite regulation after PH and SL**	1	rno-29a	0,78	TET1, VEGFA, IGF1
**C. miRNAs deregulated after SL but not following PH**	1	rno-miR-708	1,55	NRAS, KRAS, PPARA
	2	rno-miR-511	1,47	N/A
	3	rno-miR-224	1,46	SMAD4, SP7, HOXD10
	4	rno-miR-337	1,42	NLK, MET, CDK6
	5	rno-miR-484	0,61	BCL2, IRS2, PHKB

The five miRNAs that showed the strongest expression changes from each group in Table 1 were included for putative target analysis using the TargetScan rat algorithm (http://www.targetscan.org; Release 6.2, June 2012). The table provides a selection of putative targets. The ranking of microrna expression changes was performed according to the microarray data from Table 1 considering significant expression changes at all time points. For group A expression changes in the PH but not the SL group were used (since these were considered more relevant to the biological mechanisms of liver regeneration). PH – partial hepatectomy, SL – sham laparotomy, N/A – not available.

### Influence of anesthesia on miRNA expression in the liver

**Figure 1:**
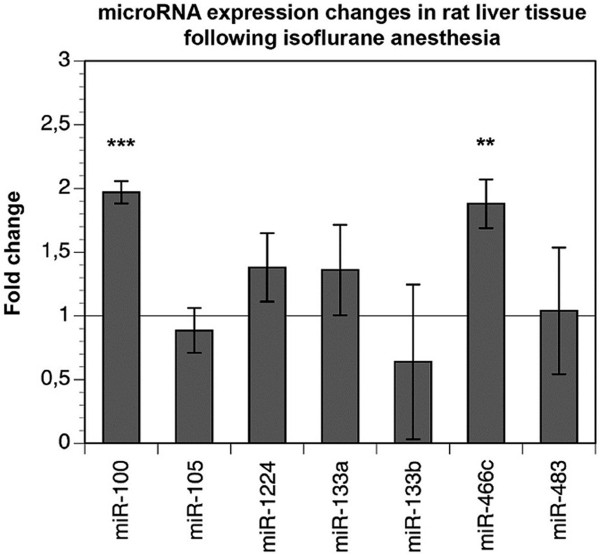
**miRNA expression in the liver of rats 24 hours after isoflurane anesthesia.** The effect of anesthesia on miRNA expression in the liver was investigated by qRT-PCR using samples of untreated animals vs. samples taken 24 hours following 45 minutes of isoflurane anesthesia and pain medication. Relative miRNA expression ratios (normal liver was considered to be 1) were normalized against stably expressed U6 small nucleolar RNA (Student’s t-test, **p < 0.01, ***p < 0.001).

We further examined the effect of inhalative isoflurane anesthesia on the expression of the miRNAs that were found to be equally deregulated following PH and SL. Out of the 10 miRNAs found to be affected, we were only able to investigate seven miRNAs since expression levels for rno-miR-383, rno-miR-598-5p, and rno-miR-628 were too low for qRT-PCR (CT cycles > 40) in rat liver tissue. Out of these miRNAs, two (rno-miR-100 and rno-miR-466c) were significantly upregulated following isoflurane anesthesia compared to untreated animals (1.97 fold and 1.88 fold expression change, respectively; p < 0.01 and p = 0.001). The other investigated miRNAs showed no significant expression changes compared to liver from non-treated animals (Figure 1).

### Surgically induced miRNAs in plasma samples

**Figure 2:**
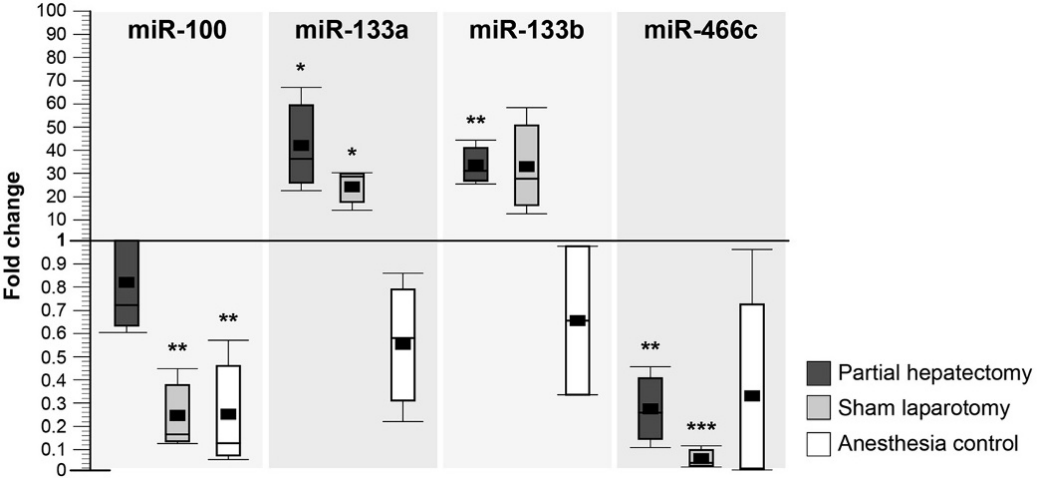
**miRNA plasma levels in rats 24 hours after partial hepatectomy, sham operation, or isoflurane anesthesia.** miRNA levels in plasma samples of Wistar rats 24 hours after partial hepatectomy (PH), sham laparotomy (SL), or anesthesia only (AN). Measurements were performed with qRT-PCR (n = 3). The results were normalized to untreated animals and are displayed in fold changes (miRNA levels in plasma samples from untreated animals was considered to be 1; Student’s t-test, *p < 0.05, **p < 0.01, ***p < 0.001). Statistically significant differences were not found 24 hours after the procedures between the PH- and SL-groups (Oneway anova, p > 0.05).

Based on these findings, we investigated whether similar miRNA expression differences were detectable in plasma samples after PH, SL or anesthesia only. Out of the similarily regulated miRNAs found in the microarray screening, only rno-miR-100, rno-miR-133a, rno-miR-133b, and rno-miR-466c were reproducibly detected in all investigated plasma samples. The expression levels of rno-miR-100 and rno-miR-466c were similarly downregulated following PH, SL, and anesthesia only compared to non-treated animals (Figure 2). Downregulation of rno-miR-100 was statistically significant in the SL- and AN-group, while rno-miR-466c downregulation was significant in the PH- and SL-group. The expression of both rno-miR-133a and rno-miR-133b was similarly increased in plasma samples after PH and SL, with a 42-fold (p = 0.04) and 24-fold change (p = 0.01) for miR-133a, respectively. The expression of both miRNAs was not increased following anesthesia only. The investigated miRNAs displayed no significant variation between the PH- and SL- groups 24 hours after treatment.

## Discussion

miRNAs are important regulators of posttranscriptional gene expression, and there is a growing body of evidence showing that miRNAs are involved in the regulation of hepatocyte proliferation and liver regeneration [20]. As PH is a common model to investigate miRNA expression changes during liver regeneration, it is important to consider the side effects of the surgical procedure itself in order to control for miRNAs that are deregulated but not directly associated with liver regeneration. We found a similar deregulation of 10 miRNAs in liver tissue following PH and SL. Only one miRNA (rno-miR-466c) was similarly regulated in the anesthesia control group; therefore the observed miRNA expression changes following PH and SL are most likely caused by the surgical procedures and/or the subsequent (inflammatory) tissue response. Importantly, these changes were also detectable in plasma samples. These findings highlight the critical need for appropriate control groups in surgical miRNA studies.

The most deregulated miRNAs (according to the strength of expression changes considering the microarray data from Table 1) are listed in Table 2. In order to identify possible molecular pathways in which these miRNAs are implicated we provide TargetScan analysis data. The putative targets of the identified miRNAs include a lot well-known regulators of hepatic growth, cell cycle control, and signal transduction, such as IGF1, SMAD4, IRS2, and SP3, among others. The identification and molecular confirmation of miRNA-mediated regulation of these putative targets needs to be performed in future investigations.

Sham operation controls are commonly performed in the miRNA analyses of other organ systems [21–23]; however, this procedure is apparently not a standard procedure in studies investigating miRNA expression changes during liver regeneration. To date, eight comparable experimental miRNA studies using a rat PH model have been published [6–10, 24–26]. Surprisingly, in three of these studies, sham operation controls were not performed at all [24–26]. Kren et al. investigated the association of mRNAs as well as miRNAs to free, membrane-, and cytoplasmic polysomes 3, 6, and 24 hours following 70% partial hepatectomy [24]. Their microarray data indicate that miRNA associations to the polysome populations changed significantly after PH (compared to non-resected liver) and they suggest that these changes might be associated with the regenerative process. However, since they did not conduct sham operation controls, the possibility of some significant changes that might have been caused by laparotomy alone exists. Chen et al. performed small liver graft transplantations in male Lewis rats using 45%, 75%, and 95% of native liver volume and 50% partial hepatectomy [25]. Although a strict cut-off was chosen (upregulation of at least 200% and downregulation of at least 50%), microarray expression changes were only applicable to native controls. No sham operation was performed, which may be a confounding factor regarding miRNA deregulation found in their analyses. Zhang et al. used PH as a traumatic liver injury model and analyzed circulating miRNAs in the serum of operated rats to determine specific biomarkers, but they also did not perform sham operations [26]. They detected 21 overexpressed miRNAs in serum 24 hours after PH. Two of these miRNAs, miR-133a and miR-133b, were also found in our analyses. Consistently, we observed a significant upregulation of both miRNAs after PH and SL in plasma samples. However, in our analyses the hepatic tissue expression of both miRNAs was significantly reduced. Our findings, therefore, suggest that the upregulation of miR-133a/b in plasma samples might be a consequence of surgical stress or trauma rather than being specific to the hepatectomy. This notion is further supported by the fact that miR-133a/b are highly expressed in muscle tissue [27, 28] and their increased expression levels in the plasma might likely be the result of muscle trauma during and after laparotomy. In general, the origin and function of extracellular miRNAs are still controversial. While circulating miRNAs are likely to be by-products of cell apoptosis or tissue injury [29], there is also evidence for active miRNA transport to the extracellular space via exosomes, indicating either clearance or specific physiological functions [30].

In addition to the role of surgical stress, anesthesia might also be a confounding factor in experimental surgery causing independent miRNA expression changes. Therefore, we performed anesthesia controls in our study, and we identified miR-100 and miR-466c to be deregulated in liver tissue and plasma samples of these animals. While hepatic tissue miR-466c was consistently upregulated after PH, SL, and AN, plasma miR-466c showed lower expression levels only following PH and SL. Furthermore, tissue miR-100 was downregulated after PH and SL but upregulated after AN, while plasma miR-100 was lower expressed after SL and AN but not after PH. These findings illustrate that plasma and tissue miRNA expression levels do not necessarily have to correlate (e.g. miRNA-466c) and that independent and differential, even counteracting, effects of surgery and anesthesia might add up to a very complex picture (e.g. tissue miRNA-100 is downregulated by SL, which encompasses surgery and anesthesia, but upregulated by AN alone). As isoflurane is known to alter gene expression of liver, kidney, and heart [31], and metamizole and buprenorphine interact with enzymes of the cytochrome P450 superfamily [32, 33], it is likely that perioperative procedures such as inhalative anesthesia and pain medication may have a considerable effect on hepatic miRNA expression changes. It should be noted at this point that different analgesic regimens were used in the different groups in our study. Both the PH- and the SL-group received buprenorphine. However, in the AN-group, buprenorphine was not given, since we observed respiratory depression/apnea under this treatment without major surgery. Therefore, the results of the AN-group exclusively highlight the effects of isoflurane and metamizole analgesia on miRNA expression changes. Moreover, we did not monitor drinking water and metamizole consumption, which is a further potential confounder of our results. As the animals did not have access to drug-free drinking water, the relative level of ingestion of metamizole should have been similar in all animals. However, therapeutic drug monitoring (i.e. measurement of isoflurane, buprenorphine, and metamizole blood levels) would be the best choice to exclude any potential confounding effects of anesthetics or analgesics on the results of surgical miRNA studies.

Another limitation of our study is its purely descriptive nature. However, it was not our intention to investigate the function of surgically induced miRNAs, but to analyze factors confounding miRNA expression screenings. It should be noted that we did not include female rats and that other rat strains (e.g. Sprague–Dawley rats) could show different results [34]. Furthermore, we can only speculate on either the mechanisms causing the observed miRNA deregulation or on its possible functional implications. However, as we also found miRNA expression changes in mice kidneys at various time points after sham laparotomy in a previous study [ [35]; data not shown], this effect might be neither species nor organ specific but represents a more general challenge for surgical miRNA studies. Moreover, miRNA expression changes following surgical interventions are not only limited to organs or tissues but can also be detected in the circulation (e.g. plasma). This should especially be considered when designing interventional clinical studies using circulating miRNAs as diagnostic biomarkers [36, 37]. In both settings the need to control for unspecific confounders is of outstanding importance. However, it is naturally difficult to use appropriate controls, such as sham operations, in a clinical setting. Therefore, animal model studies require even more sophisticated controls to allow for a more meaningful translation into the clinical setting.

## Conclusion

We show that miRNAs are significantly deregulated in liver tissue due to sham operation and anesthesia only. miRNA expression changes following surgical interventions are not only limited to organs or tissues but can also be detected in the circulation (e.g. plasma). As we also observed miRNA expression changes in mice kidneys at various time points after sham laparotomy, this effect might be neither species nor organ specific but represents a more general challenge for surgical miRNA studies. Therefore, we strongly emphasize to include appropriate control groups in surgical miRNA studies so as not to generate false-positive results.
